# Cytomegaloviral Infections in Recipients of Chimeric Antigen Receptor T-Cell Therapy: An Observational Study With Focus on Oncologic Outcomes

**DOI:** 10.1093/ofid/ofae422

**Published:** 2024-07-18

**Authors:** Fareed Khawaja, Sairah Ahmed, Swaminathan P Iyer, Joseph Sassine, Guy Handley, Rishab Prakash, Tracy VanWierren, Jennifer Jackson, Anna Zubovskaia, Jeremy Ramdial, Gabriela Rondon, Krina K Patel, Amy Spallone, Ella J Ariza-Heredia, Victor Mulanovich, Georgios Angelidakis, Ying Jiang, Roy F Chemaly

**Affiliations:** Division of Internal Medicine, Department of Infectious Diseases, Infection Control and Employee Health, The University of Texas MD Anderson Cancer Center, Houston, Texas, USA; Division of Cancer Medicine, Department of Lymphoma/Myeloma, The University of Texas MD Anderson Cancer Center, Houston, Texas, USA; Division of Cancer Medicine, Department of Stem Cell Transplantation and Cellular Therapy, The University of Texas MD Anderson Cancer Center, Houston, Texas, USA; Division of Cancer Medicine, Department of Lymphoma/Myeloma, The University of Texas MD Anderson Cancer Center, Houston, Texas, USA; Division of Internal Medicine, Department of Infectious Diseases, Infection Control and Employee Health, The University of Texas MD Anderson Cancer Center, Houston, Texas, USA; Infectious Diseases Section, Department of Medicine, University of Oklahoma Health Sciences Center, Oklahoma City, Oklahoma, USA; Division of Internal Medicine, Department of Infectious Diseases, Infection Control and Employee Health, The University of Texas MD Anderson Cancer Center, Houston, Texas, USA; Division of Infectious Disease and International Medicine, Department of Medicine, Morsani College of Medicine, University of South Florida, Tampa, Florida, USA; Division of Cancer Medicine, Department of Lymphoma/Myeloma, The University of Texas MD Anderson Cancer Center, Houston, Texas, USA; Division of Internal Medicine, Department of Infectious Diseases, Infection Control and Employee Health, The University of Texas MD Anderson Cancer Center, Houston, Texas, USA; Division of Internal Medicine, Department of Infectious Diseases, Infection Control and Employee Health, The University of Texas MD Anderson Cancer Center, Houston, Texas, USA; Division of Internal Medicine, Department of Infectious Diseases, Infection Control and Employee Health, The University of Texas MD Anderson Cancer Center, Houston, Texas, USA; Division of Cancer Medicine, Department of Stem Cell Transplantation and Cellular Therapy, The University of Texas MD Anderson Cancer Center, Houston, Texas, USA; Division of Cancer Medicine, Department of Stem Cell Transplantation and Cellular Therapy, The University of Texas MD Anderson Cancer Center, Houston, Texas, USA; Division of Cancer Medicine, Department of Lymphoma/Myeloma, The University of Texas MD Anderson Cancer Center, Houston, Texas, USA; Division of Internal Medicine, Department of Infectious Diseases, Infection Control and Employee Health, The University of Texas MD Anderson Cancer Center, Houston, Texas, USA; Division of Internal Medicine, Department of Infectious Diseases, Infection Control and Employee Health, The University of Texas MD Anderson Cancer Center, Houston, Texas, USA; Division of Internal Medicine, Department of Infectious Diseases, Infection Control and Employee Health, The University of Texas MD Anderson Cancer Center, Houston, Texas, USA; Division of Internal Medicine, Department of Infectious Diseases, Infection Control and Employee Health, The University of Texas MD Anderson Cancer Center, Houston, Texas, USA; Division of Internal Medicine, Department of Infectious Diseases, Infection Control and Employee Health, The University of Texas MD Anderson Cancer Center, Houston, Texas, USA; Division of Internal Medicine, Department of Infectious Diseases, Infection Control and Employee Health, The University of Texas MD Anderson Cancer Center, Houston, Texas, USA

**Keywords:** cytomegalovirus, chimeric antigen receptor T cell therapy, immunocompromised host

## Abstract

**Background:**

Patients with B-cell lymphoma and acute lymphoblastic leukemia (ALL) who receive chimeric antigen receptor T-cell (CAR-T) therapy may experience clinically significant cytomegalovirus infection (CS-CMVi). However, risk factors for CS-CMVi are not well defined. The aims of our study were to identify risk factors for CS-CMVi and the association between CS-CMVi and nonrelapse mortality (NRM) in lymphoma and ALL patients after CAR-T therapy.

**Methods:**

We performed a retrospective single-center cohort analysis of CAR-T recipients between January 2018 and February 2021 for treatment of lymphoma and ALL. We collected data on demographics, oncologic history, CAR-T therapy–related complications, and infectious complications within 1 year of therapy.

**Results:**

Of 230 patients identified, 22 (10%) had CS-CMVi. At 1 year following CAR-T therapy, 75 patients (33%) developed relapsed disease and 95 (41%) died; NRM at 1 year was 37%. On Cox regression analysis, Asian or Middle Eastern race (adjusted hazard ratio [aHR], 13.71 [95% confidence interval {CI}, 5.41–34.74]), treatment of cytokine release syndrome/immune effector cell–associated neurotoxicity syndrome with steroids (aHR, 6.25 [95% CI, 1.82–21.47]), lactate dehydrogenase at time of CAR-T therapy (aHR, 1.09 [95% CI, 1.02–1.16]), and CMV surveillance (aHR, 6.91 [95% CI, 2.77–17.25]) were independently associated with CS-CMVi. CS-CMVi was independently associated with NRM at 1 year after CAR-T therapy (odds ratio, 2.49 [95% CI, 1.29–4.82]).

**Conclusions:**

Further studies of immunologic correlatives and clinical trials to determine the efficacy of prophylactic strategies are needed to understand the role of CS-CMVi and post–CAR-T mortality.

## Teaser

Clinically significant cytomegalovirus infection (CS-CMVi) after chimeric antigen receptor T-cell (CAR-T) therapy is common. CS-CMVi was an independent risk factor for nonrelapse mortality at 1 year after CAR-T therapy for patients with lymphoma and acute lymphoblastic leukemia.

Chimeric antigen receptor T-cell (CAR-T) therapy has greatly changed the management of patients with hematologic malignancies, specifically those with relapsed or refractory lymphoma and or acute lymphoblastic leukemia (ALL) [1]. Initially, CAR-T therapies were approved for third-line therapy and more recently have been approved as second-line therapy, with ongoing clinical studies to assess the efficacy as first-line therapy [2–4]. For patients with B-cell lymphoma with relapse of disease within 12 months, survival is greater after CAR-T therapy compared to autologous transplantation [3, 5, 6]. CAR-T therapy is often associated with cytokine release syndrome (CRS) or immune effector cell–associated neurotoxicity syndrome (ICANS); patients often require treatment with tocilizumab, steroids, or anakinra to mitigate these complications [7]. Additionally, CAR-T therapy may lead to hypogammaglobinemia due to B-cell hypoplasia, with subsequent higher rates of infections [6–9]. Infection is one of the most common direct causes of nonrelapse mortality (NRM) after CAR-T therapy [10]. This risk is increased after treatment of CRS and/or ICANS with tocilizumab or steroids, with advanced age at the time of CAR-T therapy, and with multiple lines of therapy prior to CAR-T therapy [7, 9, 11]. Most reported viral infections following CAR-T therapy are from respiratory viruses and cytomegalovirus (CMV) [9].

CMV infection (CMVi) is common in solid organ transplant and hematopoietic cell transplant (HCT) recipients and is a major cause of posttransplant morbidity and mortality [12–15]. The introduction of letermovir for primary CMV prophylaxis in HCT recipients has been associated with a reduction in clinically significant CMVi (CS-CMVi) and in NRM after transplantation [12]. Among patients with hematologic malignancies, CS-CMVi is an uncommon complication [16–19], but when CS-CMVi occurs in lymphoma patients, overall mortality can be as high as 29% [19, 20].

Several studies have reported CS-CMVi and CMV end-organ disease in CAR-T recipients [8, 21, 22]. Approximately 22% of patients may develop CS-CMVi requiring antiviral therapy following CAR-T therapy [21, 22]. Risk factors for CS-CMVi after CAR-T therapy include steroid use and the type of CAR-T therapy [21, 23]. In a study of patients who underwent CAR-T therapy, overall survival was lower in those with CS-CMVi compared to those without CS-CMVi [22]. However, many of these studies included a small sample size and lacked a comprehensive assessment of risk factors associated with CS-CMVi. Further evaluation of risk factors, known or novel, for CMV infections unique to CAR-T therapy recipients is essential, especially as new therapies for improvement of post–CAR-T complications and outcomes are emerging. This is of particular interest for potential new immunosuppressive strategies for prevention and management of CRS and ICANS [24]. Additionally, better understanding the cause of death of CAR-T therapy recipients with CS-CMVi may underscore the benefits of some preventive strategies for CMV such as primary prophylaxis. Therefore, the goal of our study was to identify risk factors for CS-CMVi in lymphoma and ALL patients who underwent CAR-T therapy and to determine the association between CS-CMVi and NRM in CAR-T recipients.

## METHODS

### Study Design

We performed a retrospective analysis to include all patients who underwent a commercially available CAR-T therapy between January 2018 and February 2021 for treatment of underlying lymphoma or ALL at our center. During this period, tisagenlecleucel (Kymriah), brexucabtagene autoleucel (Tecartus), and axicabtagene ciloleucel (Yescarta) became commercially available. Lisocabtagene maraleucel (Breyanzi) was approved in February 2021 and was not included in our study. We collected data regarding demographics, oncologic history, CAR-T therapy–related complications, and infectious complications up to 1 year after treatment. Our outcomes of interest included CS-CMVi and NRM. We defined CS-CMVi as CMV viral load ≥500 IU/mL and required antiviral therapy or the development of CMV end-organ disease as defined by Ljungman et al [25].

### Management of CRS/ICANS

For all CAR T-cell recipients, CRS and ICANS were assessed twice daily for the duration of the hospitalization after infusion. Grading was performed using preexisting guidelines [26]. For CRS grade 1 or more, tocilizumab was recommended solely. For CRS grade 2 or more and ICANS grade 1 or more, steroids are added in combination with tocilizumab. If patients were refractory to treatment with tocilizumab and steroids, anakinra was added for a total of 7 days.

### Infection Prevention and CMV Surveillance Protocols for CAR-T Recipients

Patients undergoing CAR-T infusion at our center received prophylaxis to prevent herpes simplex or varicella zoster reactivation (acyclovir or valacyclovir) and *Pneumocystis jirovecii* pneumonia (sulfamethoxazole-trimethoprim, atovaquone, or dapsone) up to 6–12 months if the patient remains in remission. During the immediate post–CAR-T phase, patients received antifungal agents for the first 1–3 months, and those who developed neutropenia received antibacterial agents such as levofloxacin to prevent neutropenic fever. The choice of antifungals would vary between non-mold-active agents in patients without CRS/ICANS (fluconazole or echinocandins) or mold-active agents for patients who underwent treatment for CRS/ICANS (posaconazole or isavuconazole).

Patients who developed neutropenia or grade 3/4 CRS or ICANS underwent CMV surveillance. In brief, these patients underwent weekly CMV DNA polymerase chain reaction (PCR) determination in plasma samples up to 1 month from CAR-T infusion. If the CMV viral load was ≥500 IU/mL or patients had signs and symptoms of CMV end-organ disease, antiviral therapy with valganciclovir, ganciclovir, or foscarnet was recommended; treatment was deferred to the treating physician. CMV PCR testing was performed after the surveillance period or in patients in whom surveillance was not recommended at the discretion of the treating physician for workup of acute febrile episodes. Assessment for CMV end-organ disease was performed at the discretion of the treating physician. CMV serostatus testing prior to CAR-T therapy and CMV surveillance were not routinely performed until April 2019, when internal guidelines were developed and implemented.

### Data Collection and Statistical Analysis

All variables were entered into our institutional REDCap database. Oncologic data, including complications such as CRS and ICANS within 30 days of therapy, were collected using the MD Anderson cellular therapy database. Other variables were collected through manual review of patients’ electronic medical records. A waiver of consent was granted by our institutional review board.

We compared the patients with CS-CMVi and those without any CMV infection, and patients with CMV viremia of unclear significance were excluded from all CS-CMVi analyses. Fisher exact test and Wilcoxon rank-sum test were used to compare categorical and continuous variables, respectively. These methods were also used to compare patients with and without NRM at 1 year after CAR-T therapy. Cox proportional hazards regression model was used to identify the independent risk factors for CS-CMVi. First, univariate Cox regression analysis was performed for each variable. Then variables with *P* value ≤.20 from univariate analyses were selected to construct the initial multivariate Cox regression model, and the full model was reduced to the final model using the backward elimination procedure so that all the variables remaining in the final model had *P* values <.05. Some variables were treated as time-dependent variables in the Cox regression analysis, such as prolonged neutropenia, the highest recorded CRS and ICANS grades after CAR-T therapy, treatment of CRS or ICANS (including steroids, tocilizumab, and anakinra), and different coinfections after CAR-T therapy. A similar Cox regression analysis with time-dependent variables including CS-CMVi status was performed to identify the independent risk factors for NRM within 1 year after CAR-T therapy as well, and patients with relapse of their underlying malignancy were excluded from the analysis. Last, Kaplan-Meier method was used to estimate the survival curves in patients with and without CS-CMVi for overall mortality and NRM, respectively, and CS-CMVi was treated as a time-dependent variable in this analysis [27]. The Kaplan-Meier survival curves were compared using Cox regression analysis for time-dependent variables. All the tests were 2-sided tests with a significance level of .05. The data analyses were performed using IBM SPSS Statistics version 15 (IBM Corporation, Armonk, New York) and SAS version 9.4 (SAS Institute, Cary, North Carolina) software.

## RESULTS

### Patients’ Characteristics

We identified 230 patients who underwent CAR-T therapy for lymphoma or ALL over 2 years at our center. The median age for our cohort was 60 years (range, 6–89 years), and patients were predominantly male (69%) and White (64%). Patients received CAR-T therapy after a median of 4 lines of therapy (range, 1–13), with most patients having refractory (77%) and/or relapsed (68%) disease. Most of our patients had diffuse large B-cell lymphoma as an indication for CAR-T therapy (70%) and received axicabtagene ciloleucel (89%). Bacterial and fungal infections, respectively, were diagnosed in 57% and 8% of our patients during up to 1 year of follow-up. The patients’ demographic and clinical characteristics are noted in Table 1.

**Table 1.** Characteristics of Chimeric Antigen Receptor T-Cell Therapy Recipients With and Without Clinically Significant Cytomegalovirus Infection

Variable	Total (n = 230)	No CMV Infection (n = 179)	Any CMV Reactivation (n = 51)	*P* Value^a^	CS-CMVi (n = 22)	*P* Value^a^
Age at time of infusion, y, median (range)	60 (6–89)	59 (6–89)	61 (19–85)	.49	61 (20–79)	.62
Sex						
Female	71 (31)	47 (26)	24 (47)	.01	13 (59)	<.01
Male	159 (69)	132 (74)	27 (53)		9 (41)	
Ethnicity						
African American	18 (8)	13 (7)	5 (10)	.56	1 (5)	1.0000
Asian	7 (3)	1 (<1)	6 (12)	<.01	5 (23)	<.01
White	148 (64)	123 (69)	25 (49)	.01	9 (41)	.01
Non-White Hispanic	44 (19)	36 (20)	8 (16)	.55	4 (18)	1.00
Middle Eastern	8 (3)	2 (1)	6 (12)	<.01	3 (14)	<.01
Other	4 (2)	3 (2)	1 (2)	1.00	0 (0)	1.00
Declined to answer	1 (<1)	1 (<1)	0 (0)	1.00	0 (0)	1.00
Smoking status						
Active smoker	4 (2)	3 (2)	1 (2)	.82	0 (0)	.59
Former smoker	73 (32)	59 (33)	14 (27)		5 (23)	
Nonsmoker	152 (66)	116 (65)	36 (71)		17 (77)	
Unknown status	1 (<1)	1 (<1)	0 (0)		0 (0)	
Comorbidities at time of CAR-T therapy						
Hypertension	84 (37)	61 (34)	23 (45)	.19	9 (41)	.63
Chronic kidney disease, stage 2–5	16 (7)	9 (5)	7 (14)	.05	1 (5)	1.00
End-stage renal disease	4 (2)	4 (2)	0 (0)	.58	0 (0)	1.00
Diabetes mellitus	37 (16)	25 (14)	12 (24)	.13	5 (23)	.34
HIV	0 (0)	0 (0)	0 (0)		0 (0)	
Heart failure	9 (4)	6 (3)	3 (6)	.42	1 (5)	.56
COPD/asthma	9 (4)	8 (4)	1 (2)	.69	1 (5)	1.00
Interstitial lung disease	0 (0)	0 (0)	0 (0)		0 (0)	
Autoimmune disease	8 (3)	8 (4)	0 (0)	.20	0 (0)	.60
Lines of treatment prior to CAR-T therapy, median (range)	4 (1–13)	4 (1–13)	5 (1–11)	.0028^a^	4 (1–10)	.4479
Anti-CD20 mAb therapy prior to CAR-T therapy	220 (96)	171 (96)	49 (96)	1.0000	21 (95)	1.0000
Refractory disease prior to CAR-T therapy	178 (77)	140 (78)	38 (75)	.5735	18 (82)	1.0000
Relapsed disease prior to CAR-T therapy	156 (68)	123 (69)	33 (66)	.7351	18 (82)	.6387
Prior HCT						
Total	59 (26)	47 (26)	12 (24)	.86	3 (14)	.30
Autologous	52 (88)	44 (25)	8 (16)	Ref	2 (9)	Ref
Allogeneic	7 (11)	3 (2)	4 (8)	.04	1 (5)	.15
Indication for CAR-T therapy						
ALL	11 (5)	8 (4)	3 (6)	.83	2 (9)	.34
DLBCL	161 (70)	127 (71)	34 (67)		17 (77)	
High-grade B-cell lymphoma	17 (7)	14 (8)	3 (6)		0 (0)	
Mantle cell lymphoma	2 (1)	2 (1)	0 (0)		0 (0)	
Primary mediastinal lymphoma	9 (4)	7 (4)	2 (4)		1 (5)	
Follicular lymphoma	30 (13)	21 (12)	9 (18)		2 (9)	
IPI for lymphoma at time of CAR-T therapy, median (range)	2 (0–5)	2 (0–5)	3 (0–5)	.13	3 (1–5)	.011
LDH at time of CAR-T therapy, U/L, median (range)	283 (126–3582)	291 (126–3582)	255 (128–2390)	.93	411 (144–2390)	.025
Type of CAR-T therapy						.78
Tisagenlecleucel	23 (10)	19 (11)	4 (8)	.62	3 (14)	
Brexucabtagene autoleucel	2 (1)	2 (1)	0 (0)		0 (0)	
Axicabtagene ciloleucel	205 (89)	158 (88)	47 (92)		19 (86)	
Prolonged neutropenia post–CAR-T therapy^b^	35 (15)	24 (13)	11 (22)	.18	6 (29)	.10
Hypogammaglobinemia within 1 y after CAR-T	93 (40)	67 (37)	26 (51)	.11	8 (36)	1.00
HLH or MAS within 1 y after CAR-T	31 (9)	14 (8)	7 (14)	.24	5 (23)	<.01
Highest recorded CRS after 30 d from infusion						
Peak, median (range)	1 (0–4)	1 (0–4)	2 (0–4)	.10	2 (0–4)	<.01
None to grade 2	200 (87)	158 (88)	42 (82)	.34	14 (64)	<.01
Grade 3 to 4	30 (13)	21 (12)	9 (18)		8 (36)	
Highest recorded ICANS after 30 d from infusion						
Peak, median (range)	1 (0–4)	1 (0–4)	3 (0–4)	<.01	3 (0–4)	<.01
None to grade 2	143 (62)	119 (66)	24 (47)	<.01	7 (32)	<.01
Grade 3 to 4	87 (38)	60 (34)	27 (53)		15 (68)	
Treatment of CRS or ICANS						
Steroids	127 (55)	92 (51)	35 (69)	.04	19 (86)	<.01
Tocilizumab	147 (64)	111 (62)	37 (73)	.19	19 (86)	.03
Anakinra	12 (5)	5 (3)	7 (14)	<.01	5 (23)	<.001
Cumulative dose of steroids, mg/kg/day, median (range)	0 (0–9.3)	0 (0–9.3)	1.3 (0–8.5)	<.01	2 (0–8.5)	<.001
Doses of tocilizumab, median (range)	1 (0–4)	1 (0–4)	1 (0–4)	.06	2 (0–4)	<.001
CMV serostatus prior to CAR-T therapy						
Nonreactive	31 (13)	29 (16)	2 (4)	<.01	1 (5)	.05
Reactive	73 (32)	46 (26)	27 (57)		11 (50)	
Not performed	126 (55)	104 (58)	22 (43)		10 (45)	
CMV preemptive surveillance performed post–CAR-T therapy	34 (15)	13 (7)	20 (39)	<.0001	9 (41)	<.001
Coinfections						
HHV-6 reactivation	13 (6)	5 (3)	8 (16)	.0019	4 (18)	.0097^a^
Bacterial infection within 1 y	130 (57)	95 (53)	35 (68)	.0772	18 (82)	.0123^a^
Fungal infection within 1 y	19 (8)	12 (7)	7 (14)	.1453	4 (18)	.0809
Relapse	75 (33)	64 (36)	11 (22)	.06	4 (18)	.15
1-y mortality	95 (41)	68 (38)	27 (53)	.08	17 (77)	<.01
1-y nonrelapse mortality^c^	57 (37)	38 (33)	19 (48)	.13	14 (79)	<.01

Data are presented as No. (%) unless otherwise indicated.

Abbreviations: ALL, acute lymphoblastic leukemia; CAR-T, chimeric antigen receptor T cell; CMV, cytomegalovirus; COPD, chronic obstructive pulmonary disease; CRS, cytokine release syndrome; CS-CMVi, clinically significant cytomegalovirus infection; DLBCL, diffuse large B-cell lymphoma; HCT, hematopoietic cell transplantation; HIV, human immunodeficiency virus; HLH, hemophagocytic lymphohistiocytosis; HHV-6, human herpesvirus 6; ICANS, immune effector cell–associated neurotoxicity syndrome; IPI, international prognostic index; LDH, lactate dehydrogenase; mAb, monoclonal antibody; MAS, macrophage activation syndrome.

^a^Univariate performed using the cohort with no CMV reactivation as the control group.

^b^Neutrophil count <1000 cells/mL for ≥14 days.

^C^Patients with relapse excluded from calculation (n = 155).

CMV serologies were available for 104 patients (45%); of those, 73 (70%) were reactive prior to CAR-T therapy. CMV viremia was observed in 51 patients (22%); of those, 22 (10%) had CS-CMVi. Among the 31 patients who were CMV seronegative, 2 developed CMV viremia; 1 had CS-CMVi. Patients with CS-CMVi received antiviral therapy at a median viral load of 915 IU/mL (range, <34.5–100 651 IU/mL). Patients who were started on antiviral therapy at a viral load of <500 IU/mL (n = 4) had suspected CMV end-organ disease (3 for CMV pneumonitis and 1 for encephalitis) but were not stable enough to undergo invasive testing for confirmation. A total of 7 patients (3%) were diagnosed with CMV end-organ disease. Further details of CMV-related characteristics are noted in Tables 1 and 2.

**Table 2.** Characteristics of Chimeric Antigen Receptor T-Cell Therapy Recipients With Clinically Significant Cytomegalovirus Infection

Variable	CS-CMVi (n = 22)
Time from CAR-T therapy to CS-CMVi, d, median (range)	17 (0–343)
Initial viral load, IU/mL, median (range)	137 (<34–78 300)
Peak viral load, IU/mL, median (range)	915 (<34–100 651)
CMV end-organ disease, No. (%)	7 (33)
Use of valganciclovir/ganciclovir, No. (%)	15 (68)
Use of foscarnet, No. (%)	14 (64)

Abbreviations: CAR-T, chimeric antigen receptor T cell; CMV, cytomegalovirus; CS-CMVi, clinically significant CMV infection.

**Figure 1.**
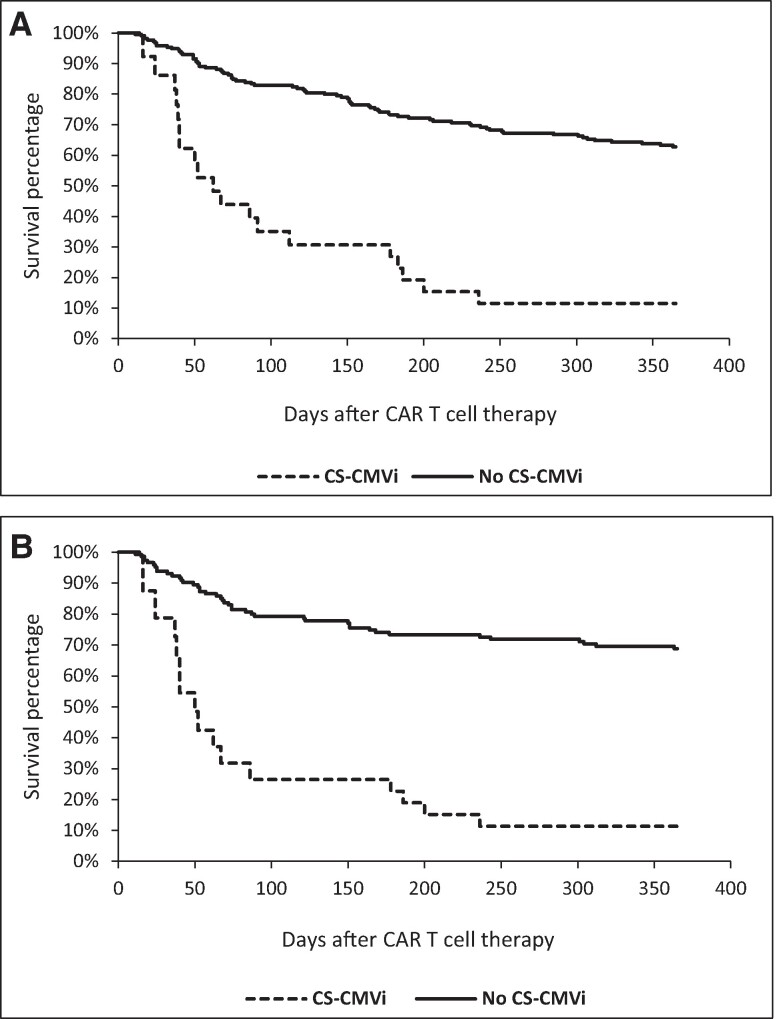
Kaplan-Meier curves illustrating survival differences between CAR T cell therapy recipients with and without CS-CMVi after 1 year. Patients with CS-CMVi had significantly worse overall survival (*P* < .001) (*A*), and relapse-free survival (*P* < .001) (*B*). Note: Patients' CS-CMVi status was treated as a time-dependent variable in the Kaplan-Meier analysis. Abbreviations: CMV, cytomegalovirus; CS-CMVi, clinically significant CMV infection; CAR, chimeric antigen receptor.

One year following CAR-T therapy, 75 patients developed relapsed disease (33%) and 95 patients died (41%). The NRM at 1 year was 37% (Table 1). We compared overall survival in CAR-T therapy recipients with and without CS-CMVi, and both survival (Figure 1*A*) and NRM (Figure 1*B*) outcomes were better in patients without CS-CMVi. Additionally, bacterial infections and human herpesvirus 6 reactivation were more often observed in patients with CS-CMVi (Table 1).

### Independent Risk Factors for CS-CMVi

On univariate analysis comparing patients with CS-CMVi (n = 22) to patients without any CMV infection (n = 179), sex, Asian and Middle Eastern race, increased international prognostic index (IPI) score for lymphoma and lactate dehydrogenase (LDH) level at time of CAR-T therapy, development of hemophagocytic lymphohistiocytosis (HLH) or macrophage activation syndrome (MAS) within 1 year of CAR-T therapy, high-grade CRS or ICANS (3 or 4) within 30 days of CAR-T therapy, and treatment of CRS/ICANS with steroids or anakinra were associated with CS-CMVi (Table 1). On Cox regression, Asian or Middle Eastern race (compared to other races), LDH at time of CAR-T therapy, treatment of CRS/ICANS with steroids, and CMV surveillance were independently associated with CS-CMVi (Table 3).

**Table 3.** Cox Proportional Hazards Regression Model of Independent Risk Factors for Clinically Significant Cytomegalovirus Infection in Chimeric Antigen Receptor T-Cell Therapy Recipients

Independent Risk Factors	aHR	(95% CI)	*P* Value
Race			
Asian or Middle Eastern	13.71	(5.41–34.74)	<.0001
Other^a^	Reference		
LDH at time of CAR-T therapy (U/L) (every 100-unit increase)	1.09	(1.02–1.16)	.011
Treatment of CRS or ICANS with steroids^b^	6.25	(1.82–21.47)	.004
CMV surveillance	6.91	(2.77–17.25)	<.0001

Abbreviations: aHR, adjusted hazard ratio; CAR-T, chimeric antigen receptor T cell; CI, confidence interval; CMV, cytomegalovirus; CRS, cytokine release syndrome; ICANS, immune effector cell–associated neurotoxicity syndrome; LDH, lactate dehydrogenase.

^a^Other races include African American, White, Hispanic, and any races other than Asian and Middle Eastern.

^b^The variable was treated as a time-dependent variable in the analysis.

### Independent Risk Factors for NRM

On univariate analysis, hypertension, elevated IPI score for lymphoma or LDH level at the time of CAR-T therapy, prolonged neutropenia (absolute neutrophil count of <1000 cells/mL for ≥14 days), HLH or MAS after CAR-T therapy, high-grade CRS or ICANS (3 or 4) within 30 days of CAR-T therapy, treatment of CRS/ICANS with steroids and/or tocilizumab, and CS-CMVi or bacterial infections within 1 year after CAR-T therapy were associated with NRM at 1 year (Table 4). On Cox regression analysis, hypertension, elevated IPI or LDH of ≥300 IU/L at time of CAR-T therapy, HLH/MAS after CAR-T therapy, fungal infection within 1 year of CAR-T therapy, and CS-CMVi were independently associated with NRM (Table 5).

**Table 4.** Comparison of Chimeric Antigen Receptor T-Cell Therapy Recipients Without Relapse Who Survived or Died After 1-Year Follow-up (Nonrelapse Mortality)

Variable	Survived (n = 97)	Died (n = 58)	*P* Value
Age at time of CAR-T therapy, y, median (range)	59 (8–86)	61 (6–89)	.1482
Sex			1.0000
Female	34 (35)	20 (34)	
Male	63 (65)	38 (66)	
Ethnicity			.9455
African American	7 (7)	5 (9)	
Asian	3 (3)	1 (2)	
White	63 (65)	38 (66)	
Hispanic	19 (20)	10 (17)	
Middle Eastern	3 (3)	2 (3)	
Other	1 (1)	2 (3)	
Declined to answer	1 (1)	0 (0)	
Smoking status			.4937
Active smoker	3 (3)	0 (0)	
Former smoker	32 (33)	17 (29)	
Nonsmoker	61 (63)	41 (71)	
Unknown status	1 (1)	0 (0)	
Comorbidities			
Hypertension	33 (34)	30 (52)	.0422
Chronic kidney disease, stage 2–5	5 (5)	4 (7)	.7285
End-stage renal disease	1 (1)	0 (0)	1.0000
Diabetes mellitus	13 (13)	12 (21)	.2632
HIV	0 (0)	0 (0)	
Heart failure	5 (5)	3 (5)	1.0000
COPD/asthma	1 (1)	2 (3)	.5563
Interstitial lung disease	0 (0)	0 (0)	
Autoimmune disease	5 (5)	1 (2)	.4115
Lines of chemotherapy prior to CAR-T, median (range)	3 (1–13)	4 (2–10)	.0526
Anti-CD20 mAb therapy prior to CAR-T	93 (96)	53 (91)	.2960
Refractory disease prior to CAR-T	74 (76)	45 (78)	1.0000
Relapsed disease prior to CAR-T	74 (76)	41 (71)	.5971
Prior HCT	30 (31)	12 (21)	.1937
Indication for CAR-T therapy			.2661
ALL	4 (4)	5 (8)	
DLBCL	66 (68)	42 (72)	
High-grade B-cell lymphoma	7 (7)	1 (1)	
Mantle cell lymphoma	1 (1)	0 (0)	
Primary mediastinal lymphoma	7 (7)	1 (2)	
Transformed follicular lymphoma	12 (12)	9 (16)	
IPI score for lymphoma at time of CAR-T therapy, median (range)	2 (0–5)	3 (0–5)	<.0001
LDH at time of CAR-T therapy, U/L, median (range)	221 (128–1514)	371 (172–3582)	<.0001
Type of CAR-T			.3189
Tisagenlecleucel	7 (7)	8 (13)	
Brexucabtagene autoleucel	1 (1)	0 (0)	
Axicabtagene ciloleucel	89 (92)	50 (86)	
Prolonged neutropenia post–CAR-T therapy^a^	9 (9)	15 (26)	.0101
Hypogammaglobinemia within 1 y after CAR-T	44 (45)	17 (29)	.0616
HLH or MAS within 1 y after CAR-T	2 (2)	14 (24)	<.0001
Highest CRS grade, median (range)^b^	1 (0–4)	0 (1–4)	.0008
Highest recorded CRS grade			.0436
None to 2	86 (89)	44 (76)	
3–4	11 (11)	14 (24)	
Highest ICANS grade, median (range)^b^	1 (0–4)	3 (0–4)	.0002
Highest recorded ICANS grade			.0007
None to 2	69 (71)	25 (43)	
3–4	28 (29)	33 (57)	
Treatment of CRS or ICANS			
Steroids	45 (46)	43 (74)	.0008
Tocilizumab	55 (57)	47 (81)	.0027
Anakinra	4 (4)	8 (14)	.0577
Cumulative dose of steroids, mg/kg/d, median (range)	0 (0–5.7)	1.3 (0–9.3)	<.0001
Doses of tocilizumab, median (range)	1 (0–4)	1 (0–4)	.0019
CMV serology			.2766
Nonreactive	13 (13)	5 (9)	
Reactive	31 (32)	26 (44)	
Not performed	53 (55)	27 (47)	
Preemptive surveillance for CMV	11 (11)	10 (17)	.3367
Infections			
Any CMV reactivation	21 (22)	19 (33)	.1341
CS-CMVi within 1 y	4 (4)	14 (24)	.0002
CMV end-organ disease	2 (2)	5 (8)	.1010
Any viral infection within 1 y	35 (35)	26 (44)	.3107
Bacterial infection within 1 y	46 (47)	39 (67)	.0198
Fungal infection within 1 y	11 (11)	9 (16)	.4670

Data are presented as No. (%) unless otherwise indicated.

Abbreviations: ALL, acute lymphoblastic leukemia; CAR-T, chimeric antigen receptor T cell; CMV, cytomegalovirus; COPD, chronic obstructive pulmonary disease; CRS, cytokine release syndrome; CS-CMVi, clinically significant cytomegalovirus infection; DLBCL, diffuse large B-cell lymphoma; HCT, hematopoietic cell transplantation; HIV, human immunodeficiency virus; HLH, hemophagocytic lymphohistiocytosis; ICANS, immune effector cell–associated neurotoxicity syndrome; IPI, international prognostic index; LDH, lactate dehydrogenase; mAb, monoclonal antibody; MAS, macrophage activation syndrome.

^a^Neutrophil count <1000 cells/mL for ≥14 days.

^b^Continuous variable.

**Table 5.** Cox Proportional Hazards Regression Model of Independent Risk Factors for Nonrelapse Mortality in Chimeric Antigen Receptor T-Cell Recipients

Independent Risk Factors	aHR	(95% CI)	*P* Value
Hypertension	1.94	(1.12–3.35)	.018
IPI for lymphoma at time of CAR-T therapy			<.0001
0–3	Reference		
4–5	3.16	(1.77–5.62)	
LDH at time of CAR-T therapy, U/L (every 100-unit increase)	1.13	(1.08–1.18)	<.0001
HLH or MAS within 1 y after CAR-T therapy	2.40	(1.18–4.88)	.016
Fungal infection within 1 y^a^	3.19	(1.39–7.31)	.006
CS-CMVi within 1 y^a^	2.49	(1.29–4.82)	.007

Abbreviations: aHR, adjusted hazard ratio; CAR-T, chimeric antigen receptor T cell; CI, confidence interval; CS-CMVi, clinically significant cytomegalovirus infection; HLH, hemophagocytic lymphohistiocytosis; IPI, international prognostic index; LDH, lactate dehydrogenase; MAS, macrophage activation syndrome.

^a^The variable was treated as a time-dependent variable in the analysis.

## DISCUSSION

Our study identified multiple independent risk factors of CS-CMVi in patients with lymphoma and ALL undergoing CAR-T therapy; these include Asian or Middle Eastern race, treatment of CRS/ICANS with steroids or anakinra, and CMV surveillance. We also noted that 10% of our patients developed CS-CMVi following CAR-T therapy. Of importance, CS-CMVi was independently associated with NRM within 1 year after CAR-T therapy. Last, we identified other risk factors for NRM within 1 year of CAR-T therapy, including hypertension, high IPI and LDH at the time of CAR-T therapy, HLH/MAS after CAR-T therapy, and fungal infections.

In prior studies, the use of steroids for treatment of CRS and/or ICANS as well as age were risk factors noted for CMV reactivation [21–23]. This is similar to findings in HCT and solid organ transplant recipients, as treatment of graft-versus-host disease or graft rejection with immunosuppressive therapy is associated with CS-CMVi [28]. This is possibly due to steroid-associated reduction in CMV-specific lymphocytes, allowing latent CMV to reactivate in HCT recipients [29]. Kampouri et al correlated reduced CMV cell-mediated immunity to corticosteroid use for >3 days at the time of CAR-T therapy and up to 4 weeks after therapy [23]. In our study, we identified additional unique risk factors for CS-CMVi, such as LDH levels prior to CAR-T therapy and being of Asian or of Middle Eastern race.

LDH and IPI are markers of disease burden prior to lymphoma treatment [30]. Previous studies have assessed the utility of LDH and IPI to predict outcomes after CAR-T therapy [31, 32]. These markers have been correlated with poor survival and increased risk of ICANS post–CAR-T infusion [31, 32]. We noted similar correlations between LDH and IPI, and NRM. In addition, an elevated LDH was associated with increased risk of CS-CMVi in our study. It is unclear whether CS-CMVi is due to increased use of immunosuppressive therapy for CRS or ICANS in patients with high disease burden or more related to lymphoma disease burden. Regardless, IPI and LDH prior to CAR-T can potentially be used to determine patients at high risk for CS-CMVi. Future studies may consider targeting these patients for CMV surveillance and/or prophylactic strategies.

CMV preemptive therapy has been used in conjunction with or without CMV prophylaxis to prevent CS-CMVi and end-organ disease in solid organ transplant and HCT recipients [33]. Compared to antiviral CMV prophylaxis, patients receiving CMV preemptive therapy have lower rates of drug-related toxicity and lower rates of late CMVi following transplantation [34–36]. By its nature, CMV preemptive therapy increases the rates of CMV viremia detection once implemented successfully [37]. However, CMV preemptive therapy is not associated with decreased NRM following transplantation, and with the introduction of letermovir, many transplant centers have now opted for antiviral CMV prophylaxis after allogeneic HCT who are recipient CMV seropositive [38]. In our study, CMV surveillance was associated with increased detection of CS-CMVi as expected and we also described an association between CS-CMVi and NRM following CAR-T therapy. On the other hand, due to the low incidence of CMV end-organ disease, we were unable to determine if CMV surveillance was associated with reduction of CMV end-organ disease. Whether antiviral CMV prophylaxis would be more favorable in terms of long-term survival in CAR-T recipients when compared to preemptive CMV surveillance needs to be determined in future clinical trials. On the other hand, CAR-T therapy recipients at high risk for progression to CMV end-organ disease may benefit from surveillance; yet determining when to initiate anti-CMV therapy is unclear.

Although infection is a common direct cause of death following CAR-T therapy [7, 10], the impact of CS-CMVi on mortality has not been well understood in this patient population as CMV end-organ disease is uncommon and not often a direct cause of death. Among transplant recipients, CS-CMVi has been associated with NRM [39], with improvement noted after the introduction of letermovir for CMV primary prophylaxis [12]. This association could be explained in part by the detrimental indirect effects of CMV reactivation on the immune system and subsequent worse outcomes [40]. Previously, Chen et al reported a reduced overall survival in patients with CMV reactivation following CAR-T therapy [22]. The authors noted that among patients with CMV reactivation, the most common cause of death was progression or relapse of underlying lymphoma (64%). Due to their study's small sample size, the authors were unable to determine if CMV reactivation was associated with NRM or increased rates of relapse following CAR-T therapy. In our study, we found that CS-CMVi was independently associated with NRM at 1 year after CAR-T therapy on multivariate analysis. However, it is unclear if higher NRM was mediated in part by CMV-associated immune dysregulation with subsequent increased risk for bacterial and fungal infections in CAR-T recipients.

Our study has multiple limitations inherent to the study design. We could not determine a temporal association with CS-CMVi and NRM despite our robust analysis. Another limitation is that our findings could not be extrapolated to patients with multiple myeloma who underwent CAR-T therapy as prior studies noted different incidence of CMVi associated with recipients of idecabtagene vicleucel (Abecma) [23]. Last, some of our patients did not undergo CMV surveillance for preemptive therapy or CMV serostatus testing as we included patients who underwent CAR-T therapy prior to the development of infection prevention guidance.

In conclusion, our study highlights novel risk factors associated with CS-CMVi following CAR-T therapy for patients with underlying lymphoma and ALL. More importantly, we found an association between CS-CMVi and NRM. Further prospective trials with immunologic correlatives are needed to better characterize the relationship between CMV and NRM. Additionally, dedicated prophylactic trials in CAR-T recipients at high risk of CMV infections are desirable to determine their impact on CMV and oncologic outcomes.

## Notes

***Acknowledgments.*** Editorial support was provided by Bryan Tutt, Scientific Editor, Research Medical Library at MD Anderson Cancer Center

***Financial support.*** This work was supported by Merck & Co. (Merck Investigator Studies Program number 60369).
